# Abnormal Peripheral Neutrophil Transcriptome in Newly Diagnosed Type 2 Diabetes Patients

**DOI:** 10.1155/2020/9519072

**Published:** 2020-04-22

**Authors:** Qiuqiu Lin, Wenzhi Zhou, Yanfei Wang, Juan Huang, Xiaoyan Hui, Zhiguang Zhou, Yang Xiao

**Affiliations:** ^1^Department of Metabolism & Endocrinology, The Second Xiangya Hospital, Central South University, Changsha, Hunan 410011, China; ^2^National Clinical Research Center for Metabolic Diseases, Changsha, Hunan, China; ^3^Key Laboratory of Diabetes Immunology (Central South University), Ministry of Education, Changsha, Hunan 410011, China; ^4^State Key Laboratory of Pharmaceutical Biotechnology, The University of Hong Kong, Hong Kong, China

## Abstract

**Aim:**

There are increasing evidence demonstrating that neutrophil-mediated inflammation plays a role in the etiology of type 2 diabetes. However, the molecular mechanisms by which neutrophils contribute to type 2 diabetes remain largely unknown. The aim of the present work was to identify possible changes in circulating neutrophils to better elucidate neutrophil involvement in human type 2 diabetes.

**Methods:**

Patients newly diagnosed with type 2 diabetes (*n* = 5) and age- and sex-matched healthy controls (*n* = 5) were recruited. Neutrophils were purified from type 2 diabetes patients and controls, and RNA sequencing (RNA-seq) was used for comprehensive transcriptome analysis. Differentially expressed genes (DEGs) were screened, and Gene Ontology (GO) and KEGG pathway enrichment analyses were performed. Real-time polymerase chain reaction (qPCR) was used for validation in external samples of type 2 diabetes patients (*n* = 8) and healthy controls (*n* = 8).

**Results:**

Gene expression analysis showed that, compared with neutrophils from healthy controls, there were 1990 upregulated DEGs and 1314 downregulated DEGs in neutrophils from type 2 diabetes patients. GO analysis demonstrated that the DEGs were mainly involved in myeloid leukocyte activation, T cell activation, adaptive immunity, and cytokine production. The top 20 enriched KEGG pathways included the cytokine-cytokine receptor interaction pathway, NF-*κ*B signaling pathway, cell adhesion molecules, and chemokine signaling pathway. Furthermore, qPCR of genes related to neutrophil activation revealed that the expression of SELL, SELP, CXCR1, and S100A8 was significantly increased in neutrophils from type 2 diabetes patients compared with that in neutrophils from controls.

**Conclusions:**

Our study reveals an abnormal activation of circulating neutrophils at the transcriptome level in type 2 diabetes patients. These findings suggest a potential involvement of neutrophil dysfunction in the pathologic process of type 2 diabetes and provide insight into potential therapeutic targets for type 2 diabetes.

## 1. Introduction

Low-grade inflammation is a common component in type 2 diabetes, particularly in the development of obesity-related insulin resistance [1]. Neutrophils are the most abundant type of white blood cell and are reported as active players in inflammation of obesity-related insulin resistance [2]. Additionally, neutrophil count, a marker of subclinical inflammation, has been shown to significantly increase in type 2 diabetes compared with healthy subjects [3–5]. Neutrophil-lymphocyte ratio significantly increases in prediabetes and diabetes and may be a predictive marker for prediabetes and diabetes mellitus [6]. Furthermore, several large-scale prospective studies demonstrated that the neutrophil count could be used as a predictor of the incidence of type 2 diabetes [7], suggesting the potential role of neutrophils in the development of type 2 diabetes.

Neutrophils eliminate extracellular pathogens by multiple strategies, including phagocytosis, degranulation to release lytic enzymes, and neutrophil extracellular traps (NETs), which are formed through a unique cell death process that is clearly different from both apoptosis and necrosis, termed “NETosis” [8–10]. However, improper activation of neutrophils may lead to tissue damage during exaggerated inflammatory responses [11]. Neutrophils from patients with type 2 diabetes reportedly produce more superoxide and cytokines [12, 13], and neutrophils from type 2 diabetes patients are more susceptible than those from healthy controls to PMA-induced NETosis [14]. Neutrophil serine proteases, which are crucial components of NET, including neutrophil elastase (NE) and proteinase 3 (PR3), have been shown to participate in the initiation of insulin resistance and type 2 diabetes [2, 15]. NE treatment elicits insulin resistance and glucose intolerance in mice, while neutrophil elastase deficiency results in improved tissue inflammation with less macrophage infiltration in adipose tissues in high-fat diet-induced obese mice [2]. Injection of recombinant PR3 alone is sufficient to induce hyperglycemia in mice, and inhibition of PR3 activity leads to an increase in glucose clearance [15]. However, the precise mechanism by which neutrophils induce type 2 diabetes remains elusive. Therefore, in this study, we aimed to identify the transcriptomic changes in circulating neutrophils from type 2 diabetes by RNA sequencing (RNA-seq) to better elucidate neutrophil involvement in type 2 diabetes.

## 2. Materials and Methods

### 2.1. Subjects

Thirteen patients with type 2 diabetes whose disease duration was less than one year were enrolled from the Second Xiangya Hospital, Central South University. The diagnosis of diabetes was based on the World Health Organization (WHO) criteria (1999). The exclusion criteria for type 2 diabetes were as follows: (1) acute infection, trauma, or surgery within one month; (2) use of antibiotics, glucocorticoids, or other immune regulators within one month; (3) severe cardiocerebrovascular, liver, kidney, or malignant disease; (4) pregnancy or lactation; (4) autoimmune diseases, such as hyperthyroidism; and (5) other types of diabetes. Thirteen gender- and age-matched controls were recruited and exhibited euglycemia using a 75 g glucose tolerance test. The exclusion criteria for the controls were the same as those above. Both patients and controls were divided into discovery group (*n* = 5 : 5) and validation group (*n* = 8 : 8) randomly.

### 2.2. Measurements

Height and weight, waist circumference, hip circumference, blood pressure, body mass index (BMI), and weight/height ratio (WHR) were obtained for all patients. Fasting venous blood samples were obtained at 8:00 am. The following biochemical parameters were assessed in fasting venous blood samples: fasting glucose, cholesterol (TC), triglycerides (TGs), fasting blood glucose, fasting C-peptide (FCP), and hemoglobin A1C (HbA1c) levels. Circulating cell counts were analyzed by the automated hematology analyzer Sysmex XE-2100. Plasma glucose was measured by a Hitachi 7170 analyzer (Boehringer Mannheim, Germany). Serum cholesterol and TG levels were measured enzymatically. Serum levels of C-peptide were assessed by the Advia Centaur System (Siemens, Munich, Germany). HbA1c was determined by liquid chromatography (Bio-Rad Laboratories, Hercules, CA).

### 2.3. Neutrophil Isolation and RNA Extraction

Human neutrophils were isolated from venous blood of patients and healthy controls by density gradient centrifugation using Ficoll-Paque Plus according to the manufacturer's protocol and then followed by positive magnetic separation for further purification using human CD16 Microbeads (Miltenyi Biotec). The cells were dissolved in TRIzol (Roche, America) in a volume of 5 − 10 × 10^6^ cells/1 mL, followed by storage at -80°C. Total RNA was extracted, and the concentration and purity of RNA were tested on a NanoDrop spectrophotometer, followed by reverse transcription using the High-Capacity cDNA Reverse Transcription Kit (Thermo Fisher Scientific, USA).

### 2.4. RNA-seq

In this study, we sequenced 5 samples from type 2 diabetes patients and 5 samples from controls on the BGISEQ-500 platform. A total of 19,718 genes were detected, averaging approximately 24.04 million reads per sample. Before downstream analyses, the raw sequencing reads, such as low-quality, polluted, and unknown base (N) reads, were filtered, followed by mapping of the clean reads to the reference genome using HISAT [16] and Bowtie2 [17]. The gene expression was calculated using a software package called RSEM [18]. The average mapping ratio to the reference genome was 92.93% (see Supplementary Tables 1 and 2), and the average mapping ratio to genes was 65.73%. According to the gene expression level, we identified differentially expressed genes (DEGs) between type 2 diabetes patients and healthy control subjects by using the DEG-seq algorithms [19]. An adjusted *P* value not greater than 0.001 and an absolute value of the log_2_ ratio greater than 1 indicated significant gene expression differences. All the samples were hierarchically clustered by the expression level of all genes. According to the DEGs, we next performed Gene Ontology (GO) classification including molecular biological function, cellular component, and biological process. With the DEGs, we performed KEGG pathway classification and functional enrichment by using phyper, which is a function of R package. We calculated the false discovery rate (FDR) for each *P* value, and the terms for which the FDR was not greater than 0.01 were defined as significantly enriched.

### 2.5. Real-Time PCR Analysis

Real-time quantitative (qPCR) was performed with Power SYBR green PCR master mix (Go Taq® qPCR, Promega Corporation, USA) on the MiniOpticon real-time PCR detection system (ViiA™ 7 Real-Time PCR System containing the Optiflex™ Optics System). All the primers used for qPCR were designed and synthesized by TSINGKE (TSINGKE Biological Technology, China). The expression of each gene was quantified as a fold change against *β*-actin by 2^-*ΔΔ*Ct^ method. Primer sequences of genes are shown in Table 1.

### 2.6. Statistical Analysis

Data are presented as the mean ± SD or median with interquartile range. Student's *t*-test was used to identify the differentially expressed groups using GraphPad Prism version 5 for Windows. Normality was determined by the Kolmogorov-Smirnov test. Data that were not normally distributed were compared by the Mann-Whitney *U* test.

## 3. Results

### 3.1. Characteristics of Participants

The anthropometric characteristics of the discovery group including 5 type 2 diabetes patients and 5 control subjects and the validation group with 8 type 2 diabetes patients and 8 control subjects are presented in Tables 2 and 3, respectively. In the discovery group, HbA1c, postprandial glucose, fasting C-peptide, systolic pressure, and diastolic blood pressure were higher in type 2 diabetes patients than in healthy controls (*P* < 0.05), and no differences were found with respect to BMI, WHR, fasting glucose, LDL-C, and TC between type 2 diabetes patients and control subjects. For the validation set, HbA1c and fasting and postprandial glucose were higher in type 2 diabetes patients than in healthy controls (*P* < 0.05).

### 3.2. Bioinformatics Analysis

**Figure 1:**
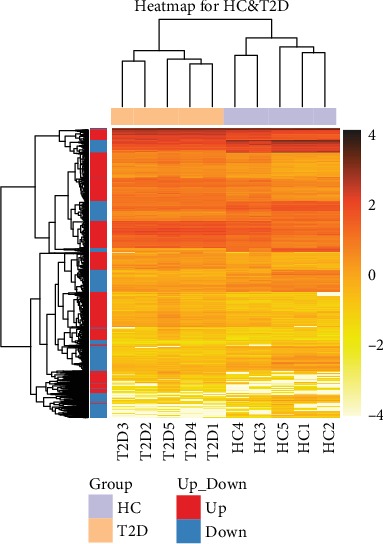
Distinct peripheral neutrophil transcriptome from newly diagnosed type 2 diabetes (T2D) patients from that of healthy controls. Heatmap shows differentially expressed genes (DEGs) in neutrophils freshly isolated from healthy controls (purple, *n* = 5) and patients with newly diagnosed T2D (orange, *n* = 5). DEGs of T2D compared with heathy controls are defined as levels of fold change ≥ 2 for upregulated genes and ≤0.5 for downregulated genes. The *X* axis shows the T2D patients and healthy controls. The *Y* axis shows DEGs. The orange color represents the log_10_ transformed gene expression level. The dark orange represents high expression level and the light orange represents low expression level.

**Figure 2:**
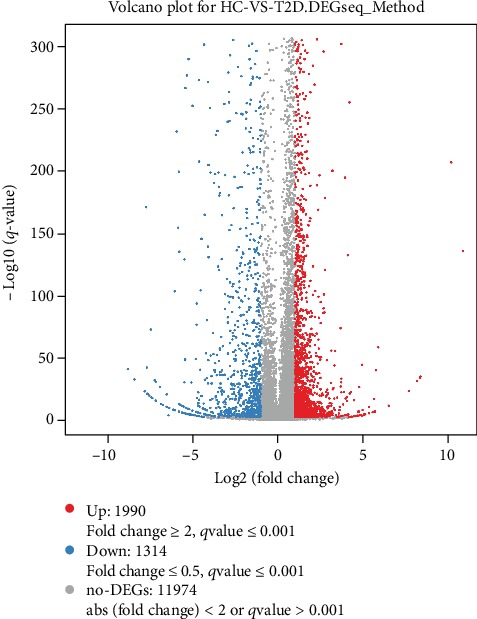
Volcano plot displaying numbers of differentially expressed genes (DEGs) in peripheral neutrophil from newly diagnosed type 2 diabetes patients compared to healthy controls. 1990 DEGs are upregulated shown in red, and 1314 DEGs are downregulated shown in blue. The *X* axis represents log_2_ transformed fold change. The *Y* axis represents -log_10_ transformed significance. Red points represent upregulated DEGs. Blue points represent downregulated DEGs. Gray points represent non-DEGs.

RNA-seq analysis of neutrophils was performed and then DEGs were identified. Compared with neutrophils from the healthy controls, there were 1990 upregulated DEGs and 1314 downregulated DEGs in neutrophils from type 2 diabetes patients. The DEG-seq algorithm was used to detect the DEGs between the two groups, and the significance of the difference was established based on the filtering criteria: fold change ≥ 2 or fold change ≤ 0.5 and adjusted *P* value ≤ 0.001 (Figures 1 and 2 and Supplementary Data 1).

### 3.3. Gene Ontology (GO) Classification

**Figure 3:**
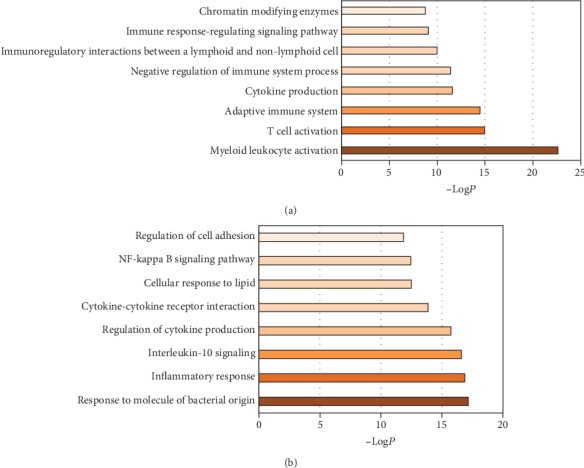
Gene Ontology (GO) analysis with the GO enrichment for differentially expressed genes (DEGs) between type 2 diabetes patients and healthy controls. DEGs of T2D compared with heathy controls are defined as levels of fold change ≥ 2 for upregulated genes and ≤0.5 for downregulated genes. (a) presents the top 8 GO categories for upregulated genes. (b) presents the top 8 GO categories for downregulated genes.

Based on these DEGs, a GO classification and a functional enrichment analysis were performed to determine the molecular functions (MM), cellular components (CC), and biological processes (BP) involved in the proteins encoded by these genes. As expected, most are important to neutrophil functioning. We discovered that GO categories of the top 8 upregulated biological functions were myeloid leukocyte activation (log*P* = −22.62), T cell activation (log*P* = −14.94), adaptive immune system (log*P* = −14.48), cytokine production (log*P* = −11.59), immune response-regulating signaling pathway (log*P* = −11.40), cytokine-mediated signaling pathway (log*P* = −10.00), immunoregulatory interactions between a lymphoid and a nonlymphoid cell (log*P* = −10.00), and immune response-regulating pathway (log*P* = −9.09) (Figure 3(a) and Supplementary Data 3). Among the downregulated GO categories, there was a close relationship with the response to molecules of bacterial origin, the biological process of the inflammatory response, interleukin- (IL-) 10 signaling, regulation of cytokine production, cytokine-cytokine receptor interaction, cellular response to lipids, and regulation of cell adhesion (Figure 3(b) and Supplementary Data 3).

### 3.4. KEGG Pathway Enrichment Analysis

**Figure 4:**
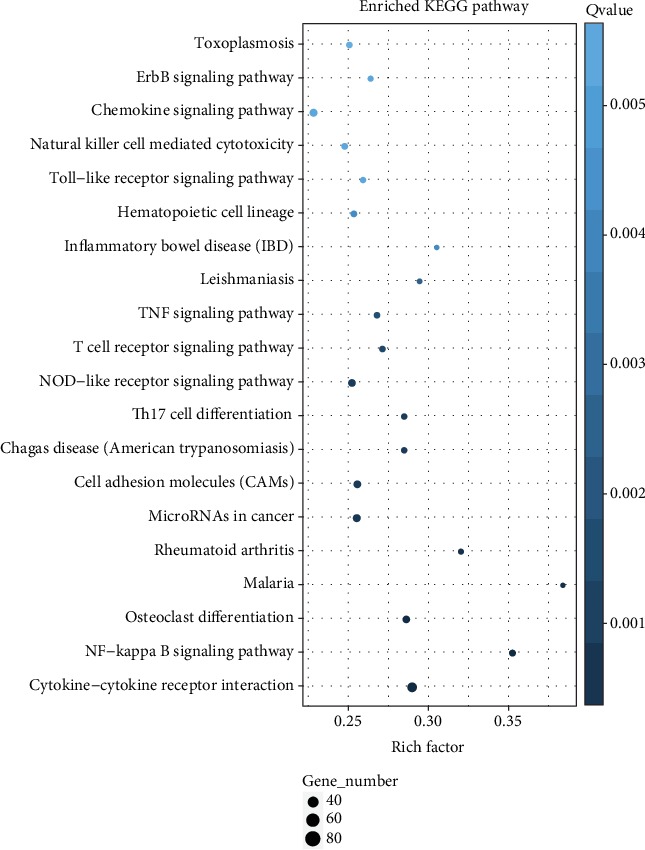
The top 20 KEGG pathways on the basis of all the differentially expressed genes (DEGs) between neutrophils from patients with type 2 diabetes and healthy controls. DEGs of T2D compared with heathy controls are defined as levels of fold change ≥ 2 for upregulated genes and ≤0.5 for downregulated genes. The *X* axis represents enrichment factor. The *Y* axis represents pathway name. The color indicates the *q* value (high: white, low: blue), and the lower *q* value indicates the more significant enrichment. Point size indicates DEG number (the bigger dots refer to larger amount). Rich factor refers to the value of enrichment factor, which is the quotient of foreground value (the number of DEGs) and background value (total gene amount). The larger the value, the more significant enrichment.

**Figure 5:**
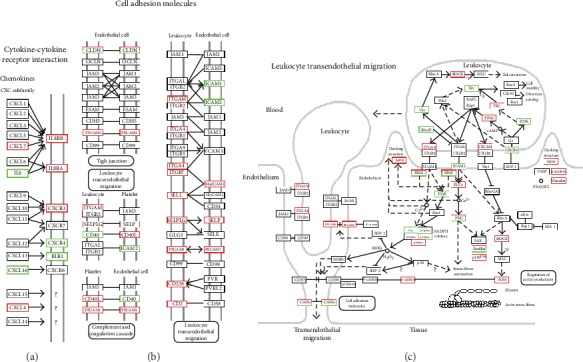
Pathway map enriched by differentially expressed genes (DEGs) of neutrophils from type 2 diabetes patients compared with healthy controls. (a) Cytokine-cytokine receptor interaction; (b) cell adhesion molecules; (c) leukocyte transendothelial migration. Red represents upregulated genes, and green represents downregulated genes.

KEGG pathway enrichment analysis of the important DEGs suggested that a wide range of biological pathways were altered in neutrophils from type 2 diabetes patients compared with controls. *P* values were used to describe the significance level of pathway enrichment. There were 318 main pathways identified, and the top 20 differential pathways were primarily involved in the cytokine-cytokine receptor interactions (*P* = 3.46∗10^−10^), NF-*κ*B signaling (*P* = 4.12∗10^−9^), tumor necrosis factor (TNF) signaling (*P* = 6.23∗10^−5^), cell adhesion molecule (CAM) signaling (*P* = 1.66∗10^−5^), Toll-like receptor signaling (*P* = 2.82∗10^−4^), and chemokine signaling (*P* = 3.25∗10^−4^) (Figure 4 and Supplementary Data 2). In the above most enriched pathways, especially cytokine-cytokine receptor interactions and cell adhesion molecule (CAM) signaling, the majority of the genes associated with the two pathways were upregulated in type 2 diabetes, such as chemokine (C-X-C motif) ligand (CXCL7, CXCL4), C-X-C chemokine receptor type (CXCR1, CXCR2), also named Interleukin 8 Receptor (IL8RA, IL8RB), as well as cell adhesion molecules integrin subunit alpha M (ITGAM), L-selectin (SELL), P-selectin (SELP), plate endothelial cell adhesion molecule-1 (PECAM1), and P-selection glycoprotein ligand-1 (PSGL-1). Pathway maps of cytokine-cytokine receptor interaction, cell adhesion molecules, and leukocyte transendothelial migration are shown in Figure 5.

### 3.5. Real-Time Quantitative PCR

**Figure 6:**
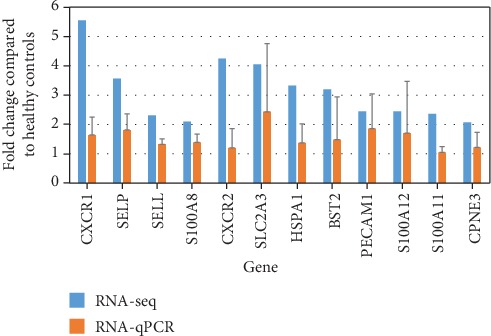
RT-qPCR validation of the RNA-seq results for a subset of 12 genes. In order to validate the RNA-seq transcriptome, 12 genes were selected from diverse biological functional categories, and RT-qPCR was performed on these genes. External samples including type 2 diabetes patients (*n* = 8) and healthy controls (*n* = 8) were selected for RNA-qPCR validation. Blue bars denote the RNA-seq fold induction values, while colored bars represent RT-qPCR fold induction values calculated using the 2^-*ΔΔ*Ct^ method, and data are presented as the mean ± SD. For RNA-qPCR validation, CXCR1, SELL, SELP, and S100A8 were significantly increased in neutrophils from type 2 diabetes patients compared with healthy controls (*P* < 0.05).

We next expanded the analysis of the purified neutrophils. In the RNA-seq results, genes were directly with neutrophil activation, like the expression of adhesion molecules like SELL, SELP, PECAM1, and related ligands or receptors, such as CXCR1, CXCR2, calcium-binding protein (S100A8, S100A11, and S100A12), bone marrow stromal cell antigen 2 (BST2), heat shock protein family A member 1A (HSPA1), and Copine 3 (CPNE 3) were increased in type 2 diabetes (Figure 6). For the validation group, we performed qPCR for these genes. As adhesion molecules, the expression of SELL and SELP in neutrophils from patients was increased compared with that in neutrophils from healthy controls (*P* = 0.030 and *P* = 0.003). In addition, CXCR1 expression was higher in neutrophils from type 2 diabetes patients than in those from healthy controls (*P* = 0.022). The levels of S100A8, which is derived mainly from neutrophils regarded as a mediator of inflammation, were higher in neutrophils from type 2 diabetes patients than in those from healthy controls (*P* = 0.019). However, comparable levels of PECAM1, CXCR2, SLC2A3, BST2, S100A11, S100A12, and CPNE3 were found in neutrophils from diabetes patients and controls (*P* > 0.05).

## 4. Discussion

Neutrophils are the first-line immune cells involved in inflammation, and circulating neutrophil counts are moderately increased in type 2 diabetes [3, 7]. However, the role of neutrophils in the pathogenesis of type 2 diabetes is largely unknown. Our study is the first to investigate the presence of DEGs and the biological functions associated with these genes in neutrophils from type 2 diabetes and healthy individuals. The study has shown that neutrophils from patients with type 2 diabetes presented increased neutrophil activation, responses to chemokines and neutrophil transendothelial cell migration at the transcriptome level.

In the current study, we used an RNA-seq dataset to assess the neutrophil gene expression changes at the transcriptome level between patients with type 2 diabetes and healthy controls. A total of 3304 DEGs were identified, including 1990 upregulated genes and 1314 downregulated genes. According to the GO analysis, myeloid leukocyte activation was the most significant among the top 20 enriched terms, which is consistent with previous studies showing increased leukocyte activation in patients with insulin resistance and type 2 diabetes [20, 21], indicating a potential role of leukocyte activation in the pathogenesis of type 2 diabetes.

Our data showed that the process of leukocyte-endothelial adhesion was activated in the analyzed neutrophils as the pathway map of leukocyte transendothelial migration shown. The adhesion genes ITGAM (CD11b), PECAM1, SELL, and SELP and the receptor PSGL-1 were upregulated in type 2 diabetes, and the mRNA levels of SELL and SELP were also increased in neutrophils from patients with type 2 diabetes, as assessed by real-time PCR; however, there was no difference in PECAM1 levels in neutrophils between patients and healthy controls. Selectins play unique roles in neutrophil recruitment by mediating recognition and adhesion between leukocytes and vascular endothelial cells. Mice lacking in L-selectin and PSGL-1 show worse neutrophil recruitment into the inflamed peritoneum than PSGL-1 knock-out mice. L-selectin (CD62L), which is expressed by most leukocytes, is involved in neutrophil trafficking [22]. P-selectin, encoded by SELP, can capture leukocytes from the circulation to the vessel wall, leading to the rolling of neutrophils [23], and polymorphisms of SELP are associated with vascular risk of type 2 diabetes [24]. The KEGG pathway analysis demonstrated that neutrophil rolling function, neutrophil activation, and adhesion were dysregulated in patients with type 2 diabetes compared with healthy controls. However, the literature also shows that CD62L is decreased in peripheral blood neutrophils in patients with diabetic microangiopathy, as assessed by flow cytometry [25]. This difference may be explained by differences between transcriptome and protein levels.

The migration of neutrophils across endothelial cells to the vascular wall is an essential step in tissue damage and the inflammatory response. Adhesion molecules mediate the adhesion of neutrophils to vascular endothelial cells. Several studies have confirmed the activation of neutrophils and elevated CD11b expression in diabetic patients [20, 21]. Moreover, it has been shown that the neutrophil-secreted enzyme NE impairs insulin signaling and increases insulin resistance. Conversely, obese mice without NE fed a high-fat diet showed improvement in insulin sensitivity [2]. Furthermore, NE has been detected in the plasma of type 2 diabetes patients who had elevated levels of glycated hemoglobin [26].

CXCR1 and CXCR2 widely exit on the cell surface of neutrophils [27]. Neutrophils expressing CXCR1/2 can be recruited to the pancreas by murine *β* cells, and macrophages produce C-X-C motif ligand 2 (CXCL2) in autoimmune diabetes [28], which plays a vital role in the early stages of diabetes. The expression of CXCR1/2 decreased after bariatric surgery in female adipose tissue [29]. Moreover, CXCR2-deficient mice are resistant to diet-induced insulin resistance and diabetes, mainly because CXCL5 blocks insulin signaling in muscle by activating the muscle Jak/STAT/SOCS pathway through the CXCR2 receptor [30]. In accordance with this finding, our RNA-seq analysis revealed that the CXCR1 and CXCR2 genes were significantly upregulated in patients with type 2 diabetes compared with healthy controls. Consistent with this result, the real-time PCR results showed that CXCR1 mRNA levels were significantly increased and that there was a tendency toward increased CXCR2 mRNA levels in type 2 diabetes.

Type 2 diabetes is associated with worse outcomes and mortality caused by infection [31] due to impaired innate immune functions, including phagocytosis, cytokine and reactive oxygen species (ROS) production, bactericidal activity, and chemotaxis [32]. In our study, the GO analysis showed that the diabetic neutrophil response to molecules of bacterial origin, such as LPS, and inflammatory response were decreased. In addition, the gene expression of cytokines/chemokines, such as CXCL2, CXCL3, CXCL5, and CXCL8, was downregulated. These data demonstrate that neutrophils from patients with diabetes may also exhibit impaired migration because of the downregulated chemotaxis, which may explain why some patients with diabetes have increased infection rates. Kuwabara et al. showed that neutrophils had an impaired response to LPS in a type 2 diabetes and obesity animal model, and neutrophils from the GK rats were not capable of migrating to the site of inflammation due to the impaired expression of adhesion proteins after LPS stimulation [32]. In addition, a defect in the chemotaxis of leukocytes in patients with diabetes has been identified, which could contribute to increased infections in these patients [33, 34], and high blood glucose induces a defective leukocyte-endothelial interaction in rats [35]. Our research is based on a small sample size, so the results may have limited generalizability. In addition the males are the majority among the participants, so our opinions may be generalizable mainly to males. Depending on the existing essay, the future research needs more representative sample and focus on making more depth analyses.

## 5. Conclusion

Our study investigated the presence of DEGs and the biological functions associated with these genes in neutrophils from type 2 diabetes patients and healthy controls. The study has shown that patients with type 2 diabetes have increased neutrophil activation, increased responses to chemokines, and increased neutrophil transendothelial cell migration at the transcriptome level. On the other hand, in patients with type 2 diabetes, neutrophil responses to molecules of bacterial origin, such as LPS, the cellular response to bacteria, and inflammatory reactions are reduced. These findings support the role of neutrophils in the pathogenesis of type 2 diabetes and provide insight into potential therapeutic targets for type 2 diabetes.

## Figures and Tables

**Table 1:** Primer sequences of forward and reverse primers.

Gene	Sense (5′ to 3′)	Antisense (3′ to 5′)
CXCR1	TCAAGTGCCCTCTAGCTGTT	TGATCTAACTGAAGCACCGGC
CXCR2	TCTGCCTAGAGCTCTGACTAC	CTGGGCTTTTCACCTGTAGGA
SELL	TCTGTTGTGATTTCCTGGCAC	CCCACCCACGTCCATATTCC
SELP	CCCAGTGTGTAAAGCTATTTCGT	GCTCCTCTCAGCATGAAACCT
PECAM1	TTTTGCCGTCTGAGTGGC	CTTGAACAGAGCAGAAGGGTCA
S100A8	AGACCTGAAGGTTCTGTTTTTCA	AGGACACTCGGTCTCTAGCA
S100A11	GCATCGAGTCCCTGATTGCT	AGGGTCCTTCTGGTTCTTTGTG
S100A12	ATTCCTGTGCATTGAGGGGTTA	TGTCAAAATGCCCCTTCCGA
SLC2A3	CGTGGAGAAAACTTGCTGCTG	TCAGAGCTGGGGTGACCTTC
HSPA1	CGCAACGTGCTCATCTTTGA	TCGCTTGTTCTGGCTGATGT
BST2	TGTCGCAATGTCACCCATCT	AGCCATTAGGGCCATCACAGT
CPNE3	GACTCCCACGAAACTCAGGT	AACATTCAGCGCCACCTTTG
*β*-Actin	GCATCCCCCAAAGTTCACAA	AGGACTGGGCCATTCTCCTT

**Table 2:** Clinical and biochemical characteristics of the study participants for RNA-seq.

	HC (*n* = 5)	T2D (*n* = 5)	*P* value
Sex (male/female)	5 (4/1)	5 (3/2)	1.000
Age (years)	43.40 ± 13.22	41.40 ± 7.50	0.776
BMI (kg/m^2^)	23.18 ± 2.21	23.63 ± 13.27	0.942
WHR	0.86 ± 0.06	0.92 ± 0.03	0.081
DBP (mmHg)	74.40 ± 5.32	81.80 ± 5.85^∗^	0.028
SBP (mmHg)	106.20 ± 8.95	124.00 ± 11.85^∗^	0.029
TG (mmol/L)	1.03 ± 0.56	1.86 ± 0.62	0.056
TC (mmol/L)	4.22 ± 0.49	5.15 ± 1.27	0.166
HDL-C (mmol/L)	1.47 ± 0.44	1.17 ± 0.14	0.210
LDL-C (mmol/L)	2.34 ± 0.54	3.42 ± 1.23	0.109
HbA1c (%)	5.52 ± 0.46	7.80 ± 2.04^∗^	0.040
Fasting BS (mmol/L)^a^	5.16 (4.88~5.36)	9.88 (5.52~10.22)	0.082
2 h postprandial BS (mmol/L)	4.88 ± 1.64	12.58 ± 5.17^∗^	0.013
Fasting C-peptide (pmol/L)	350.36 ± 90.08	707.36±207.37^∗∗^	0.008
2 h postprandial C-peptide (pmol/L)	1535.70 ± 549.43	1209.26 ± 82.11	0.225
White cell count (10^9^/L)	6.38 ± 1.35	7.70 ± 1.92	0.241
Lymphoid cell count (10^9^/L)	1.80 ± 0.47	2.61 ± 0.64	0.052
Neutrophil count (10^9^/L)	4.15 ± 1.25	4.68 ± 1.59	0.569
Mononuclear count (10^9^/L)	0.32 ± 0.12	0.31 ± 0.09	0.822

Data are expressed by mean ± SD or median (25-75th percentile). BMI: body mass index; WHR: waist to hip ratio; DBP: diastolic blood pressure; SBP: systolic blood pressure; TG: triglycerides; HDL-C: high-density lipoprotein cholesterol; LDL-C: low-density lipoprotein cholesterol; ^a^compared by the Mann-Whitney *U* test. ^∗^*P* < 0.05 compared with HC. ^∗∗^*P* < 0.01 compared with HC.

**Table 3:** Clinical and biochemical characteristics of the study participants for validation.

	HC (*n* = 8)	T2D (*n* = 8)	*P* value
Sex (male/female)	8 (6/2)	8 (6/2)	1.000
Age (years)	44.25 ± 8.41	47.63 ± 10.74	0.496
BMI (kg/m^2^)	22.40 ± 2.10	23.82 ± 2.83	0.273
WHR	0.85 ± 0.08	0.88 ± 0.05	0.388
DBP (mmHg)	75.85 ± 7.85	79.88 ± 8.01	0.316
SBP (mmHg)	121.00 ± 11.20	118.50 ± 16.45	0.733
TG (mmol/L)	1.61 ± 0.81	1.54 ± 1.58	0.903
TC (mmol/L)^a^	4.77 (4.05~4.93)	3.96 (2.50~4.56)	0.050
HDL-C (mmol/L)	1.14 ± 0.48	1.51 ± 0.92	0.332
LDL-C (mmol/L)	2.75 ± 0.36	2.07 ± 0.89	0.065
HbA1c (%)^a^	5.30 (5.20~5.55)	6.50 (6.05~7.70)^∗∗∗^	<0.001
Fasting BS (mmol/L)^a^	4.61 (4.21~5.11)	5.97 (5.14~7.65)^∗∗^	0.002
2 h postprandial BS (mmol/L)	4.88 ± 1.64	12.58±5.17^∗∗∗^	<0.001
Fasting C-peptide (pmol/L)	418.71 ± 90.49	369.44 ± 141.28	0.420
2 h postprandial C-peptide (pmol/L)	1598.80 ± 711.38	1229.03 ± 548.98	0.264
White cell count (10^9^/L)	5.97 ± 0.81	5.73 ± 0.42	0.411
Lymphoid cell count (10^9^/L)	1.97 ± 0.54	1.83 ± 0.83	0.695
Neutrophil count (10^9^/L)^a^	3.41 (3.12~3.50)	3.42 (2.88~3.68)	0.878
Mononuclear count (10^9^/L)	0.40 ± 0.08	0.30 ± 0.07	0.095

Data are expressed by mean ± SD or median (25-75th percentile). BMI: body mass index; WHR: waist to hip ratio; DBP: diastolic blood pressure; SBP: systolic blood pressure; TG: triglycerides; HDL-C: high-density lipoprotein cholesterol; LDL-C: low-density lipoprotein cholesterol; ^a^compared by the Mann-Whitney *U* test. ^∗^*P* < 0.05 compared with HC. ^∗∗^*P* < 0.01 compared with HC. ^∗∗∗^*P* < 0.001 compared with HC.

## Data Availability

The data used to support the findings of this study are available from the corresponding author upon request.

## Supplementary Materials

Supplementary 1 Supplementary Data 1: information of DEGs including gene ID, expression, and log_2_ ratio (T2D/HC).

Supplementary 2 Supplementary Data 2: information of main pathways identified in KEGG.

Supplementary 3 Supplementary Data 3: upregulated and downregulated gene annotations and identified biological processes in GO analysis.

Supplementary 4 Supplementary Table 1: RNA-seq sequence reads mapping to reference transcripts using Bowtie2. Supplementary Table 2: RNA-seq sequence reads mapping to reference genome using HISAT.
