# Supplementary material for: Abnormal Peripheral Neutrophil Transcriptome in Newly Diagnosed Type 2 Diabetes Patients
Source: J Diabetes Res. 2020 Apr 22;2020:9519072. doi: 10.1155/2020/9519072 (PMC7195634; doi:10.1155/2020/9519072)
Supplement: Supplementary 4 — Supplementary Table 1: RNA-seq sequence reads mapping to reference transcripts using Bowtie2. Supplementary Table 2: RNA-seq sequence reads mapping to reference genome using HISAT. [file 9519072.f4.docx]

[Supplementary](https://www.sciencedirect.com/science/article/pii/S0378111919308182?via=ihub#s0110) table 1 RNA-Seq sequences reads mapping to reference transcripts using Bowtie2

| Sample | Number of clean reads (mb) | Number of mapped reads (mb) | Mapped reads (%) |
| --- | --- | --- | --- |
| HC1 | 23988514 | 14795177 | 61.68 |
| HC2 | 23981056 | 14144440 | 58.98 |
| HC3 | 24030172 | 14941404 | 62.18 |
| HC4 | 23988688 | 15543063 | 64.79 |
| HC5 | 24022970 | 14436036 | 60.09 |
| T2D1 | 24066284 | 15843101 | 65.83 |
| T2D2 | 24061292 | 16526804 | 68.69 |
| T2D3 | 24070991 | 16829186 | 69.91 |
| T2D4 | 23977084 | 15583099 | 64.99 |
| T2D5 | 24055427 | 16832899 | 69.98 |

HC: heathy control; T2D: type 2 diabetes.

[Supplementary](https://www.sciencedirect.com/science/article/pii/S0378111919308182?via=ihub#s0110) Table 2 RNA-Seq sequences reads mapping to reference genome using HISAT

| Sample | Total Clean Reads | Total Mapping Ratio | Uniquely Mapping Ratio |
| --- | --- | --- | --- |
| HC1 | 23988514 | 93.97% | 82.23% |
| HC2 | 23981056 | 93.48% | 81.64% |
| HC3 | 24030172 | 93.18% | 81.76% |
| HC4 | 23988688 | 93.54% | 81.33% |
| HC5 | 24022970 | 93.29% | 81.25% |
| T2D1 | 24066284 | 92.85% | 80.92% |
| T2D2 | 24061292 | 92.92% | 80.60% |
| T2D3 | 24070991 | 92.91% | 80.68% |
| T2D4 | 23977084 | 93.07% | 81.39% |
| T2D5 | 24055427 | 93.79% | 82.08% |

HC: heathy control; T2D: type 2 diabetes.
